# Effect of Calf Gender on Milk Yield and Fatty Acid Content in Holstein Dairy Cows

**DOI:** 10.1371/journal.pone.0169503

**Published:** 2017-01-09

**Authors:** Amy V. Gillespie, James L. Ehrlich, Dai H. Grove-White

**Affiliations:** 1 Livestock Health and Welfare, School of Veterinary Science, University of Liverpool, Leahurst Campus, Chester High Road, Neston, United Kingdom; 2 Dairy Veterinarians Group, Argyle, NY, United States of America; Van Andel Institute, UNITED STATES

## Abstract

The scale of sexed semen use to avoid the birth of unwanted bull calves in the UK dairy industry depends on several economic factors. It has been suggested in other studies that calf gender may affect milk yield in Holsteins- something that would affect the economics of sexed semen use. The present study used a large milk recording data set to evaluate the effect of calf gender (both calf born and calf *in utero*) on both milk yield and saturated fat content. Linear regression was used to model data for first lactation and second lactation separately. Results showed that giving birth to a heifer calf conferred a 1% milk yield advantage in first lactation heifers, whilst giving birth to a bull calf conferred a 0.5% advantage in second lactation. Heifer calves were also associated with a 0.66kg reduction in saturated fatty acid content of milk in first lactation, but there was no significant difference between the genders in second lactation. No relationship was found between calf gender and milk mono- or polyunsaturated fatty acid content. The observed effects of calf gender on both yield and saturated fatty acid content was considered minor when compared to nutritional and genetic influences.

## Introduction

There is little demand in the UK for dairy bull calves, and consequently many are euthanased shortly after birth, representing a welfare and ethical issue for the industry.[1] The use of sexed semen in artificial insemination to avoid unwanted bull calves results in a lower conception rate than unsexed semen. Studies have estimated 8–17.9% lower conception rate in heifers, and current industry advice is to avoid its use in multiparous cows.[2–4] The economics of sexed semen use is heavily influenced by market prices.[5] If there were associations between calf gender and milk yield or composition, this could have important consequences for the value of sexed semen use.

Relationships between gender of calf born / being gestated and milk yield have been investigated, but studies to date are equivocal. Hinde et al[6] examined a data set from 1.49 million US Holsteins and found milk yield to be increased when they had given birth to a heifer calf, or were gestating a heifer calf when compared to a bull calf. Animals delivering female calves at both first and second lactations produced 454kg (2.7%) more milk than those delivering two bull calves. Canadian data agreed, but demonstrated a much smaller effect of less than 0.5%,[7] and Iranian data[8] also showed higher milk production in dams giving birth to heifers for up to four parities. The yield advantage conferred by heifer calves was only seen in the second lactation in New Zealand Holstein-Friesians,[9] and only in the first lactation in French Holstein-Friesians.[10] In contrast, Graesboll et al[11] found that bull calves conferred a milk yield advantage in a dataset from 578 Danish Holstein herds.

Sex-bias is not a new concept. In evolutionary biology, the Trivers-Willard hypothesis proposes that female mammals are able to adjust the sex of their offspring based on their own condition in order to maximise reproductive success in the next generation. Well-nourished mothers invest in sons as strong sons will produce more grandchildren, whereas daughters will produce more grandchildren than weaker sons.[12] In agreement with this theory, infant sex in people has been shown to have an effect on milk energy content, with milk produced for males being more energy dense in well-nourished mothers,[13] whilst daughters of mothers with low socioeconomic status receive higher fat milk than sons.[14]

The mechanisms by which calf sex could make a difference to milk production are not fully understood. One suggestion is that the gender of the calf *in utero* influences the endocrine control of mammogenesis. Although it is generally accepted that prolactin and placental lactogens have roles in mammogenesis and lactogenesis, the exact mechanisms for this remains the subject of debate.[15,16] This is a complex process influenced by a wide range of factors including nutrition and genetic potential, and so the role of calf gender is uncertain.[15]

Calf birthweight may influence milk production, and therefore gender could have a role via this mechanism since bull calves have larger birthweights.[17] One study has shown that larger calves are associated with greater milk production perhaps due to higher concentrations of oestrogen and placental lactogens during gestation.[18] On the contrary, Swali and Wathes[19] found that smaller calf birthweights were associated with greater milk production during gestation. It has therefore been hypothesised that gestating a larger calf causes greater partitioning of nutrients to the foetus, thus decreasing milk production. Alternatively, these results could be interpreted as high milk production in the dam predisposing to smaller calf birthweight.[19]

Giving birth to a bull calf could also reduce milk production in the subsequent lactation due to increased incidence of assisted calving.[20,21] Dystocia is associated with periparturient diseases such as metritis[21–23] and fat mobilization syndrome,[24,25] both of which could be expected to reduce milk production. The effect of assisted calving on milk production, however, was reviewed by Fourichon and others.[26] Whilst some research showed a detrimental effect following dystocia on subsequent milk production, others showed no significant loss. There may be a short term effect,[27,28] but no effect on 305 day milk yield.

Recent development and refinement of Fourier Transform Infrared (FTIR) technology has allowed cost-effective analysis of fatty acid content of milk samples collected by the Cattle Information Service (CIS) as part of their milk recording service. The percentage of fatty acids in milk is of interest for two reasons. Firstly, reducing saturated fatty acids (SFAs) and trans fatty acids in milk is believed to be preferable for cardiovascular health.[29] Dairy products account for 25–35% of SFA consumption in human nutrition.[30] High consumption of SFAs is strongly associated with poor health in people, for example atherosclerosis, obesity and coronary heart disease with lauric (12:0), myristic (14:0) and palmitic (16:0) acids considered particularly detrimental.[31,32] Dairy products are also low in protective polyunsaturated fatty acids (PUFAs) and so are considered to be more harmful to health than red meat, which is the next largest source of SFAs in human nutrition.[31] Secondly, lower SFA production in the rumen reduces methane production, with environmental benefits. Several strategies for achieving reduced methane production this have been investigated to date including dietary management and genetic potential.[33] If calf gender influenced the fatty acid composition of milk, this could influence sexed semen use in an industry drive to produce a healthier consumer product manufactured with lower environmental impact.

As demonstrated here, information regarding possible effects of calf gender on milk production is contradictory at best. Our objective was to further examine the possibility of sex-biased milk production in Holsteins using UK data. As well as energy corrected milk yields, we also examine data regarding differences in saturated, monounsaturated and polyunsaturated fatty acid composition of milk according to calf gender.

## Methods

This research did not involve human or animal participants or tissues, however it was nonetheless approved by the University of Liverpool Veterinary Research Ethics Committee. Data was extracted from The Cattle Information Service (CIS) milk recording database. Inclusion criteria were first and second lactation animals only, Holstein Friesian breed and recording milk fatty acid content. This resulted in a database of milk yield and fatty acid composition from 211,932 animals from approximately 2,000 herds. The time period covered was from when CIS started recording fatty acid measurements (5^th^ February 2013) to the date of data extraction (22^nd^ December 2014). For each animal the data contained lactation number, calving dates, gender of calf born at the start of lactation, and the following information from each milk recording: date, days in milk, yield, fat, protein, SFAs, monounsaturated fatty acids (MUFAs), PUFAs, total unsaturated fatty acids, trans fatty acids, tetradecanoic acid (C14_0), hexadecanoic acid (C16_0), octodecanoic acid (C18_0) and myristoleic acid (C14_1). Information on total unsaturated fatty acids was not used in this analysis as these values are derived from total fat and saturated fatty acid measurements. Information on individual fatty acids was not used due to inherent inaccuracies in their measurement. For all animals data regarding the gender of the calf born at onset of lactation was available. However for animals recorded in both the 1^st^ and 2^nd^ lactations, data regarding gender of calf *in utero* during the 1^st^ lactation was also available allowing the impact of this to be investigated in this sub-set.

An individual lactation curve (DIM, milkweight) was calculated for each lactation using the Milkbot® lactation model and fitting engine as described by Ehrlich.[34] Daily milk component values were estimated by linear interpolation between test points. Lactation 305-day totals were then calculated by symbolic integration of the Milkbot® equation to give 305-day Milk Yield (M305) and by summation of daily predicted milk yield times component concentration to give fat yield (F305), protein yield (P305), saturated fatty acids (SFA305), monounsaturated fatty acids (MONO305) and polyunsaturated fatty acids (POLY305). No 305-day-values were calculated if the lactation was shorter than 203 days. For those between 203 and 305 days, the last available test value was used for milk components. If the last test value was missing, an average of the data for the existing part of the lactation was used.

Data was imported in to STATA 14 (Statacorp, USA) for analysis. Lactations where calf sex was unknown were excluded, for example recorded as “unknown” or “dead calf.” Analysis included only lactations where a single heifer or single bull calf was born. The variable “CALFSEX” was generated to describe the sex of the calf born at the start of the lactation. The variable “CALFGEST” was generated to describe the sex of the calf being gestated during the lactation, thus this data was only available for Lactation 1 for animals that had reached at least day 203 of Lactation 2.

M305 was modified using the equation below to generate values for 305 day Energy Corrected Milk yield (ECM305), using fat yield (F305) and protein yield (P305) expressed as percentages.[35]

(M305*(0.383*F305%+0.242*P305%+7.832)/3.1138)

Univariable linear regression models were fitted for lactations 1 and 2 separately with the following outcome variables: ECM305, SFA305, MONO305 and POLY305. Explanatory variables were CALFSEX, calving date, and CALFGEST for lactation 1 models only. Calving date was included in the models as an explanatory variable as it is known to affect milk yield. Calving date was offered to models as a composite of four sine and cosine functions to allow modelling of seasonal periodicity if present.[36] Four time covariates (*x*_1_, *x*_2_, *x*_3_, *x*_4_) were generated as follows, where t = calving date: $\begin{array}{l} x_{1}=\sin(2\pi /365.25)\\ x_{2}=\sin(4\pi t/365.25)\\ x_{3}=\cos(2\pi t/365.25)\\ x_{4}=\cos(4\pi t/365.25) \end{array}$

Finally, multivariable linear regression models for each outcome variable were fitted for Lactation 1 and Lactation 2. Explanatory variables were CALFSEX, calving date and lactation number for all models. CALFGEST was included in all lactation 1 models, and ECM305 for the outcomes SFA305, MONO305 and POLY305.

## Results

The dataset contained 1,062,058 milk recordings relating to 211,932 animals. Following exclusions, 72,606 lactation 1 and 63,168 lactation 2 animals remained. Only 31,146 animals were eligible for analysis in models relating to lactation 1, due to missing values regarding gender of calf being gestated. There were 63,168 animals included in models relating to lactation 2, and 135,774 were available for analysis for both lactations, since CALFGEST was not used in the final model. Reasons for loss of lactations were: exclusion of twin births and gestations, unknown calf gender, and missing values for ECM305 as lactation did not reach at least 203 days.

**Fig 1:**
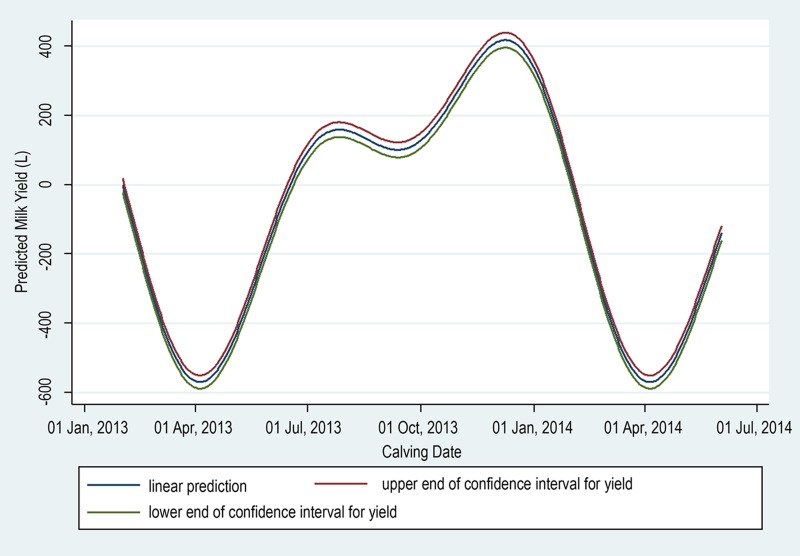
Predicted variation in ECM305 according to calving date.

The unadjusted effect of calving date on milk yield is shown in Fig 1. There is a clear milk yield advantage to calving during August to September, and December to January.

### Lactation 1

In lactation 1, giving birth to a heifer calf increased ECM305, decreased SFA305, and had no significant effect on MONO305 or POLY305. The milk yield increase was 78.1 litres, which equates to 1.0%, and the reduction in SFA305 was 0.66kg, which equates to 0.35%. Gestating a heifer had no significant effect (Table 1).

**Table 1:** Results of multivariable**linear regression models examining factors affecting ECM305, SFA305, MONO305 and POLY305 for Lactation 1

Model	Explanatory variable	Coefficient	95% CI	P-value
**Model 1**	**CALFSEX = heifer**	78.1	42.3–114.0	<0.001
**ECM305**	**CALFGEST = heifer**	29.4	-6.0–64.8	0.1
**N = 31,146**	**Baseline**	7547.9.7	7514.0–7581.8	
**Model 2**	**CALFSEX = heifer**	-0.66	-1.06–0.25	0.001
**SFA305**	**CALFGEST = heifer**	0.039	0.36–0.44	0.85
**N = 31,146**	**ECM305**	0.026	0.026–0.027	<0.001
	**Baseline**	-10.3	-11.3–9.3	
**Model 3**	**CALFSEX = heifer**	-0.041	-0.19–0.11	0.58
**MONO305**	**CALFGEST = heifer**	-0.045	-0.19–0.099	0.54
**N = 31,143**	**ECM305**	0.01025	0.0102–0.0103	<0.001
	**Baseline**	1.02	0.66–1.39	
**Model 4**	**CALFSEX = heifer**	0.0042	-0.019–0.027	0.72
**POLY305**	**CALFGEST = heifer**	-0.020	-0.042–0.0032	0.093
**N = 31,143**	**ECM305**	0.0010	0.0010–0.0010	<0.001
	**Baseline**	0.34	0.29–0.40	

Baseline = single bull born at start of first lactation and single bull being gestated.

** time variables omitted from table.

### Lactation 2

In lactation 2, giving birth to a heifer calf decreased ECM305, and had no significant effect on SFA305, MONO305 or POLY305. The milk yield decrease was 45 litres, which equates to 0.5% (Table 2).

**Table 2:** Results of multivariable**linear regression models examining factors affecting ECM305, SFA305, MONO305 and POLY305 for Lactation 2.

Model	Explanatory variable	Coefficient	95% CI	P-value
**Model 5**	**CALFSEX = heifer**	-45.0	-75.5 –-14.4	0.004
**ECM305**	**Baseline**	9008.2	8986.0–9030.3	
**N = 63,168**				
**Model 6**	**CALFSEX = heifer**	0.279	-0.066–0.62	0.113
**SFA305**	**ECM305**	0.0273	0.0272–0.0274	<0.001
**N = 63,168**	**Baseline**	-19.0	-19.8–-18.1	
**Model 7**	**CALFSEX = heifer**	0.030	-0.093–0.15	0.636
**MONO305**	**ECM305**	0.0098	0.0098–0.0099	<0.001
**N = 63,126**	**Baseline**	-0.414	-0.711–0.117	
**Model 8**	**CALFSEX = heifer**	0.0017	-0.18–0.021	0.866
**POLY305**	**ECM305**	0.000948	0.000942–0.00953	<0.001
**N = 63,126**	**Baseline**	0.170	0.123–0.218	

Baseline = single bull born at start of second lactation.

** time variables omitted from table.

## Discussion

This study showed a 1.0% milk yield advantage if a heifer was born to a primiparous dam and a 0.5% advantage if a bull was born to a second-calver. This is the first study to evaluate SFA, MUFA and PUFA content of milk in relation to calf gender. We found a small reduction in SFA produced by primiparous animals giving birth to a heifer calf (0.66kg), and no relationship between calf gender and MUFAs or PUFAs. Gestating a heifer during first lactation had no significant effect on the parameters measured.

In agreement with the present study, three previous studies have shown favourable yields when a heifer calf is born to a primiparous dam,[6–8] whilst three studies have shown dams calving bulls at the start of second lactation have higher yields.[9–11] In any case, reported effects are always marginal- the 2.7% advantage for two heifer calves born in the first two lactations found by Hinde et al6 is the largest reported by far. Other factors that affect milk yield such as mastitis[37] and lameness[38] are likely to be more important in a herd overall than calf gender.

This study did not find a statistically significant effect on milk yield of sex of calf being gestated during first lactation. This is in agreement with Hinde et al,[6] who showed that having a heifer in the first parity meant higher milk yield regardless of sex of the second calf, whilst Beavers and Doormaal[7] found giving birth to two consecutive heifer calves resulted in the greatest positive impact on milk yield. Barbat et al[10] concluded birth of a bull calf followed by a heifer calf resulted in the greatest positive impact on milk yield. In contrast, Graesboll et al[11] found birth of 2 consecutive bull calves had the greatest positive impact on milk yield.

The differences could in part be explained by differences in the milk yield data used. For example, Hess[9] used total lactational yield, calculated using the test interval method. Hinde[6] and Barbat[10] used the test day model[39] rather than predicting 305 day milk yields. Graesboll[11] adopted a farm-based approach using Wilmink curves to calculate 305 day milk yields. The Milkbot lactation model used in this study has been shown to achieve daily milk yield predictions within 0.5kg.40 Model accuracy, however, is affected by factors such as environment and genetics.[40]

For our own data, there is a potential source of bias relating to a gender imbalance in single calves born to primiparous dams: 58% were heifers whereas other studies have suggested a sex ratio approaching 50:50,[41] or even skewed towards more bull calves 53.3:46.7.[42] There are several possible explanations. Firstly, lactations were excluded from analysis if a “dead calf” or “culled calf” was recorded. It is possible that a higher proportion of these were male rather than female, however this effect was not seen in second lactation data. Secondly, there could be an effect of sexed semen use in heifers amongst the herds in this dataset. Due to the higher costs and lower conception rates from sexed semen, industry recommendations are to use sexed semen only on maiden heifers,[43] hence why this effect is not seen in second lactation. Previous studies have used more historic data describing lactations prior to widespread introduction of sexed semen use to avoid this effect. Finally, there is a possibility that primiparous cattle favour conceiving heifer calves. There is evidence of skewed birth sex ratios in other species depending on maternal condition. For example in red deer dominant females give birth to a higher proportion of males than their subordinates.[44] Dominant hinds produce higher levels of progesterone in the early days of pregnancy, and male blastocysts secrete interferon-tau earlier than females. It is proposed that maternal recognition of pregnancy in dominant hinds is therefore more likely to be successful if the blastocyst is male.[44] Factors such as this at the time of maternal recognition of pregnancy in cattle could affect calf sex.

It is possible that the use of sexed semen has affected our results. The main use for sexed semen is to breed higher genetic merit heifers,[45] (not simply more heifers), which could lead to a bias in this study as higher genetic merit heifers are selected for service with sexed semen, and therefore give birth to heifer calves. This group could be expected to have increased milk yield compared to the average, influencing study results. Furthermore, heifers calving to sexed semen may be older because conception rate to sexed semen is lower and therefore average age of conception is likely to be older.[42] Data was not available on age at first calving, but it has been shown that heifers calving for the first time at more than twenty-six months old will have higher 305 day milk yields.[46]

Despite the small reduction found in SFA in first lactation animals giving birth to a heifer calf, the magnitude of the reduction makes this finding irrelevant for human health. We did not find any relationship between calf gender and unsaturated fatty acids. Unsaturated fatty acids account for only 25–35% of total fat in milk[30] so any potential differences caused by calf gender could be expected to be smaller than those found in SFA content. There are other factors that are already well known to have substantial influence on milk fat content, particularly nutrition and genetics.[32,47,48] High starch diets are known to increase de novo synthesis of fatty acids in the mammary gland, resulting in higher concentration of SFAs in milk, whilst higher intakes of PUFAs, for example from pasture, result in higher concentration of unsaturated fatty acids in milk.[49] Similarly, dietary changes and genetic influences are known to have much greater effect on methane production in the rumen,[33] and therefore there are more viable options for reducing emissions from farms other than breeding a particular sex of calf.
